# Supplementary material for: At Which Area Level Does COVID-19 Infection Matter Most for an Individual’s Self-Rated Health? A Multilevel Fixed-Effects Model Analysis in Japan
Source: Int J Environ Res Public Health. 2022 Jul 22;19(15):8918. doi: 10.3390/ijerph19158918 (PMC9331239; doi:10.3390/ijerph19158918)
Supplement: Supplementary file 1 [file ijerph-19-08918-s001.zip › ijerph-1764293-supplementary.pdf]

QUESTIONNAIRE*

*The Japanese version is available upon request from the corresponding author.

Questionnaire about yourself

About your current life in general

Q1. Overall, on a scale from 0 for "not satisfied at all" and 10 for "very satisfied," how satisfied are you with your current life??

0 to 10 points

Q2(1) Which of the following factors has the greatest influence on your current satisfaction or dissatisfaction with your household finances and assets? Please select all that apply.

1. Total household income (including pension)
2. Total household expenditures
3. Amount of household financial assets (deposits, savings, securities, etc.)
4. Amount of household debt
5. Degree of affluence (upper, middle, or lower class)
6. Future income (expected income, pension, etc.)
7. Future burdens (expected increases in taxes, medical expenses, insurance premiums, etc.)
8. **Other (   )**

(2) Which of the following factors has the greatest influence on your current level of satisfaction or dissatisfaction with the work environment and pay? Please select all that apply.

1. Employment status (regular, non-regular, etc.)
2. Amount of salary
3. Treatment and benefits besides salary (welfare, etc.)
4. Working environment (relationship with work colleagues, level of danger, risk of death from overwork, etc.)
5. Ease of finding a job, changing jobs, or opening a business (abundance of employment opportunities)
6. Risk of unemployment, bankruptcy, business closure and so on (job stability)
7. Job satisfaction
8. Anxiety about not being able to draw a career path
9. Anxiety about future employment (e.g., life-long employment, life after retirement, fixed-term employment)

8. Other (   )

(3) What has had the greatest impact on your current level of satisfaction or dissatisfaction with your housing? Please select all that apply.

1. Total floor space of the house (its size)
2. Type of residence (owner-occupied or rented)
3. Rent and housing expenses (property tax, utilities, repairs, furniture, etc.)
4. Mortgage (including land)
5. Housing safety (earthquake resistance, fire resistance, anti-flood measures, etc.)
6. Housing comfort (insulation, soundproofing, etc.)
7. Convenience of housing (distance from convenience stores, public facilities, transportation, etc.)
8. Usability as an asset
9. Surroundings such as parks, green areas, and waterside areas
10. **Other (   )**

(4) Which of the following factors has the greatest influence on your current satisfaction or dissatisfaction with your work-life balance? Please select all that apply.

1. Working hours
2. Commuting time
3. Time spent on household chores, childcare, nursing care, and so on (outside working hours)
4. Time to play and relax with family
5. Sleeping, taking meals, bathing, etc.
6. Time that can be used freely for myself
7. **Other (   )**

(5) Which of the following factors has the greatest influence on your current satisfaction or dissatisfaction with your health? Please select all that apply.

1. time spent on daily living, including sleep, rest, sports, etc.
2. Diet (nutritional balance, caloric intake, etc.)
3. Amount of smoking and drinking
4. Stress from work and personal life
5. Opportunities for medical checkups and consulting medical institutions (ease of access to hospitals, clinics, pharmacies, etc., and types of medical departments and examinations)
6. Whether there is a doctor whom you trust
7. Expenses for maintenance and promotion of health (expenses for sports, health care services, etc.)
8. Expenses for medical care, insurance premiums, etc. (medical fees, prescription fees, insurance premiums, etc.)
9. Anxiety about future health (dementia, inability to get a doctor, etc.)
10. **Other (   )**

(6) Which of the following factors greatly affects your current level of satisfaction or dissatisfaction

with your own educational environment and standard of education? Please select all that apply.

1. My educational background
2. Ease of commuting to school (commuting time, public transportation, etc.)
3. The range of choices of higher education, cram schools, and preparatory schools (e.g., universities and graduate schools)
4. Educational institutions' responses to truancy and bullying
5. Quality of school education (e.g., how well university and college studies help students to find jobs, education that develops individuality)
6. Quality of recurrent education (e.g., useful for work)
7. Ease of use of culture courses and specialized education for working people (municipal courses, universities, colleges, etc.)
8. Education and culture expenses for my own activities
9. **Other ( )**

(7) What has had the greatest impact on your current level of satisfaction or dissatisfaction with your social relationships and community? Please select all that apply.

1. Whether you have friends and acquaintances you can rely on when you are in trouble
2. Whether there are colleagues and supervisors at work you can rely on in times of need
3. Whether or not you have friends to have fun with
4. Frequency of interaction with friends
5. Opportunities and conditions for participation in volunteer activities, community associations, neighborhood associations, etc.
6. Connections on social media (Facebook, Twitter, LINE, Instagram, etc.)
7. Anxiety about losing social ties
8. **Other ( )**

(8) Which of the following factors has the greatest influence on your current level of satisfaction or dissatisfaction regarding trust in politics, government, and the courts? Please select all that apply.

1. Judgments by politicians, government, and the courts
2. Easy-to-understand explanations by politicians, government, and the courts
3. Media coverage of politics, government, and the courts
4. Opportunities to participate in politics, government, and the courts
5. Reflecting the public's voice in politics, government, and the courts
6. **Other ( )**

(9) Which of the following factors has the greatest influence on your current level of satisfaction or dissatisfaction with the natural environment surrounding your life, such as air and water? Please select all that apply.

1. Climate (hours of sunshine, temperature, weather, etc.)

2. Air quality (air pollution, dust, dioxin, PM2.5, etc.)

3. Water quality (e.g., pollution of rivers, waterways and waterbodies)

4. Noise and vibration (e.g., noise from neighbors and aircraft, car noise, noise and vibration from construction sites)

5. Soil (dioxin, radioactivity, etc.)

6. Offensive odors (such as odors in the neighborhood and those from waste, agriculture, forestry, fishery, and industrial production).

7. Other (　　　)

(10) Which of the following greatly affects your current satisfaction or dissatisfaction with your sense of safety? Please select all that apply.

1. Dissemination of information by the government in the event of a disaster (evacuation announcements, safety confirmation, assessment of damage, etc.)

2. Post-disaster response (establishment of evacuation centers, support and assistance, reconstruction measures, etc.)

3. Deteriorating social infrastructure (tunnels, rivers, sewage systems, etc.)

4. Prevention of natural disasters (strengthening of national infrastructure, construction of levees, securing evacuation routes, etc.)

5. Prevention of traffic accidents and other accidents caused by human activities (road maintenance, measures for old drivers, etc.)

6. Risk of harm from harassment, bullying, abuse, or other crimes

7. Risk of damage from crime such as violence, theft, or arson

8. Risks related to the environment surrounding the Internet, such as leak of personal information or phishing scams

9. Risk of infection

10. Risk of terrorism

11. Other (　　　)

(11) Which of the following greatly affects your current level of satisfaction or dissatisfaction with the ease of raising children? Please select all that apply.

1. Whether or not there are people or places (parents, friends, school child care, etc.) where you can leave your child(ren) temporarily

2. Quality of childcare support services (types of services, conditions for use, consultation and support system, information on availability of daycare centers, etc.)

3. Expenses for childcare and education

4. Spouse's employment status (working hours, leave of absence/vacation, etc.)

5. Ease of taking maternity/paternity leave at work (e.g., whether or not the workplace supports childcare support)

6. Quality of school education (e.g., how well university and college studies help students to find jobs,

education that develops individuality)

7. Range of choices for higher education, cram schools, and preparatory schools

8. School and preschool responses to truancy and bullying

9. Anxiety about having children

10. I am not interested in this field

11. Other (　　　)

(12) Which of the following factors has had the greatest influence on your current level of satisfaction or dissatisfaction with the ease with which you are able to care for your family member? Please select all that apply.

1. Concerns about when you or your family members will come to need care

2. Whether there is anyone (spouse, parent, friend, etc.) who can temporarily take care of those you care for

3. A range of choices of long-term care services (home services, home-visit care, preventive care, day-care, etc.)

4. Ease of admission to long-term care facilities (number of facilities, location, capacity, admission conditions, etc.)

5. Quality of nursing care services (types of services, conditions of use, regular patrols and immediate response, counseling and support system for worries, etc.)

6. Expenses for nursing care

7. Ease of taking nursing care leave at work (e.g., whether the workplace supports nursing care)

8. Spouse's employment status (working hours, leave of absence/vacations taken, etc.)

9. New care services (e.g., care robots)

10. I am not interested in this field.

11. Other (　　　)

(13) Which of the following factors has the greatest influence on your current level of satisfaction or dissatisfaction with your enjoyment of life? Please select all that apply.

1. A good range of hobbies and objectives in life

2. Stability and fulfillment of family life

3.The possibility of taking on new challenges and working in a variety of ways (starting a business, dual jobs, side jobs, expanding mid-career hiring, returning to work after illness, injury, or childbirth, etc.)

4. Ability to participate in society and contribute to the community, or being engaged with such activities (volunteer work, community involvement, etc.)

5. Introduction and utilization of new products and services

6. The speed and difficulty of switching between digital devices (e.g., difficulty in understanding how to use the latest digital appliances and smartphones).

7. Faster and more hectic life due to information technology, etc.

8. Your own health condition

9. Status of interaction with friends

10. Job description, future prospects, working hours

11. Other (   )

Q3. On a score of 0 to 10, where 0 means "not satisfied at all" and 10 means "very satisfied," how would you rate the impact each of the following has on your current satisfaction or dissatisfaction with them?

(1) "Household finances and assets"

0 to 10 points

(2) "Work environment and pay"

0 to 10 points

(3) "Housing"

0 to 10 points

(4) "Work-life balance"

0 to 10 points

(5) "Health status"

0 to 10 points

(6) "Your own educational environment and educational level"

0 to 10 points

(7) "Social ties, including friendships and community"

0 to 10 points

(8) "Confidence in politics, government, and the courts"

0 to 10 points

(9) "The natural environment, such as the air and water around us"

0 to 10 points

(10) "Personal safety"

0 to 10 points

(11) "Ease of raising children"

0 to 10 points

(12) "Ease of caring and being cared for"

0 to 10 points

(13) "The joy and fun of life"

0 to 10 points

Q4. Please select all the following experiences you have had in the past year.

1. Married
2. Divorced
3. Bereavement of a family member

4. Unemployed or out of business
5. Housing acquired
6. Moved out
7. Infected with COVID-19
8. Major illness/injury (other than COVID-19)
9. I was subjected to harassment, bullying, abuse, etc.
10. Damaged by natural disaster
11. None that apply

- About your household and family

Q5. Please identify all the members of your household.

1. Grandparent(s) (including spouse's grandparents)
2. Parent(s) (including spouse's parents)
3. Spouse
4. Sibling(s) (including sibling(s) of spouse)
5. Child(ren)
6. Grandchild(ren)
7. **Other**
8. No other household members (only myself)

Q6. Please identify all household members who are under the age of 18.

1. Preschool child(ren)
2. Elementary school pupil(s)
3. Junior high school student(s)
4. High school student(s)
5. College, junior college, and further education college student(s)
6. University or graduate school student(s)
7. Employed
8. **Other**
9. No children under 18 years old

Now, I would like to ask you about your life under the state of emergency against COVID-19. For the purposes of this study, **"under the state of emergency"** is defined as follows:
Under the state of emergency: This refers to a period of a year and a half from April 2020, when the first state of emergency was issued, to September 2021, which was the last emergency declaration period to date. Including the period during which the declaration was lifted, this one-and-a-half-year period is uniformly described nationwide as "under the state of emergency" (from April 2020 to September 2021).

*Please answer each of the following questions by taking this 18-month period as "under the state of emergency," although it differs in some respects from the actual period of emergency declarations.

[For those with elementary school children]

Q7. Please indicate for all of the following whether (1) through (3) apply regarding your (elementary school) child's online education.

*Under the state of emergency: Please answer by taking the 18-month period from April 2020 to September 2021 as "under the state of emergency," although this period differs in part from the actual period of the declared state of emergency.

(1) Current education

(2) Education under the declared state of emergency (April 2020–September 2021)

(3) Education you would like to see continued in the future

	(1)	(2)	(3)
1. Online classes by school teachers			
2. Online tutoring by school teachers (e.g., via email)			
3. Provision of online educational materials from school to be used at home			
4. Online classes at cram schools and extracurricular schools			
5. Online tutoring (e.g., via email) at cram schools and extracurricular schools			
6. Providing online educational materials from cram schools and other extracurricular schools to be used at home			
7. Other online education			
8. No applicable online education			
9. Don't know			

[For those with junior high school students]

Q8. Please indicate for all of the following whether (1) through (3) apply regarding your (junior high school) child's online education.

*Under the state of emergency: Please answer by taking the 18-month period from April 2020 to September 2021 as "under the state of emergency," although this period differs in part from the actual period of the declared state of emergency.

(1) Current education

(2) Education under the declared state of emergency (April 2020–September 2021)

(3) Education you would like to see continued in the future

	(1)	(2)	(3)
1. Online classes by school teachers			
2. Online tutoring by school teachers (e.g., via email)			

3. Provision of online educational materials from school to be used at home			
4. Online classes at cram schools and extracurricular schools			
5. Online tutoring (e.g., via email) at cram schools and extracurricular schools			
6. Providing online educational materials from cram schools and other extracurricular schools to be used at home			
7. Other online education			
8. No applicable online education			
9. Don't know			

[For those with high school students]

Q9. Please indicate for all of the following whether (1) through (3) apply to your (high school) child's online education status.

*Under the state of emergency: Please answer by taking the 18-month period from April 2020 to September 2021 as "under the state of emergency," although this period differs in part from the actual period of the declared state of emergency.

(1) Current education

(2) Education under the declared state of emergency (April 2020 - September 2021)

(3) Education you would like to see continued in the future

	(1)	(2)	(3)
1. Online classes by school teachers			
2. Online tutoring by school teachers (e.g., via email)			
3. Provision of online educational materials from school to be used at home			
4. Online classes at cram schools and extracurricular schools			
5. Online tutoring (e.g., via email) at cram schools and extracurricular schools			
6. Providing online educational materials from cram schools and other extracurricular schools to be used at home			
7. Other online education			
8. No applicable online education			
9. Don't know			

Q10. Which of the following applies to your family members in need of care? Please answer all that apply.

1. No family members in need of care
2. Admitted to an institution

3. Trying to enter an institution, but have not yet done so
4. Resident in serviced senior housing, fee-based senior housing, group home, etc.
5. Home visitation or day care services at home
6. Family members or relatives are providing care at home (yourself as the primary caregiver)
7. Family members or relatives are taking care of the family at home (other family members are the main caregiver, and you help them)
8. Family members or relatives are providing care at home (you are not a caregiver)

[For those with family members in need of care]

Q11. During the period under the state of emergency (from April 2020 to September 2021), was there any change in the care needs of your family members who require nursing care? If there are two or more persons, please answer for the most severely ill person.

*Under the state of emergency: Please answer by taking the 18-month period from April 2020 to September 2021 as "under the state of emergency," although this period differs in part from the actual period of the declared state of emergency.

1. Improved under the state of emergency and is continuing to improve
2. Improved under the state of emergency, but now back to the original state
3. Worsened under the state of emergency, but now back to normal
4. Worsened under the state of emergency and is still worsening
5. No particular change

- About yourself

Q12. Please select only one of the following regarding your employment situation.

1. Regular employment (not on a fixed term contract and with full-time working hours)
2. Non-regular employment (part-time workers, contract workers, dispatched workers, fixed-term workers, etc., other than regular employees)
3. Director of a company, etc.
4. Self-employed (including helpers)
5. Working from home
6. Student and working part-time
7. Student, not working part-time
8. Not a student, not employed (seeking employment)
9. Not a student, not employed (not seeking employment)

(For non-regular employees and those working from home)

Q12.1. Why are you in your present occupation?

1. Because the job fits my preferences and conditions
2. Because there is no job that meets my needs and conditions (I want regular employment).
3. Because there is no job that meets my needs and conditions (I do not wish to have regular

employment)

4. Other ( )

[For those who are employed]

Q13. Please describe the type of business you work in.

1. Agriculture and forestry
2. Fishery
3. Construction
4. Manufacturing
5. Wholesale
6. Retail
7. Finance, insurance, and real estate
8. Transportation and communication
9. Electricity, gas, and water
10. Service
11. Civil servant
12. Education, learning support
13. Medical care, welfare
14. Childcare
15. **Other**

[For those who are employed]

Q14. Please indicate the work type for (1) to (3).

*Under the state of emergency: Please answer by taking the 18-month period from April 2020 to September 2021 as "under the state of emergency," although this period differs in part from the actual period of the declared state of emergency.

(1) Current work status

(2) Average work under the state of emergency (April 2020–September 2021)

(3) Future Hopes

	(1)	(2)	(3)
1. Telework (almost 100%)			
2. Mainly telework (more than 50%), combined with working in office			
3. Mainly working in office (more than 50%), with some telework			
4. Working in office (almost 100%)			
5. Occupations for which telework is not applicable			

[To student]

Q15. Regarding your own online education situation, please indicate which were true for (1) to (3).

*Under the state of emergency: Please answer by taking the 18-month period from April 2020 to September 2021 as "under the state of emergency," although this period differs in part from the actual period of the declared state of emergency.

(1) Current education

(2) Education under the declared state of emergency (April 2020–September 2021)

(3) Education you would like to see continued in the future

	(1)	(2)	(3)
1. Online classes by school teachers			
2. Online tutoring by school teachers (e.g., via email)			
3. Provision of online educational materials from school to be used at home			
4. Online classes at cram/preparatory schools and extracurricular schools			
5. Online tutoring (e.g., via e-mail) at cram/preparatory schools and extracurricular schools			
6. Providing online educational materials from cram/preparatory and extracurricular schools			
7. Other online education			
8. No applicable online education			

Q16. What is your highest level of education? If you are currently in school, please select the type of school you are attending as "expected."

1. Junior high school/expected
2. High school/expected
3. Specialized further education college/expected
4. College and junior college/expected
5. University/Expected
6. Graduate school/Expected

- Health and medical care

Q17. How do you feel about your own health? Please indicate the condition of your health for (1) and (2).

*Under the state of emergency: Please answer by taking the 18-month period from April 2020 to September 2021 as "under the state of emergency," although this period differs in part from the actual period of the declared state of emergency.

(1) Current health condition

(2) Average health status under the state of emergency (April 2020–September 2021)

	(1)	(2)

1. Good		
2. Reasonably good		
3. Normal		
4. Not so good		
5. Not good		

Q18. Do you have any worries or stress in your daily life? Please indicate the level of stress for (1) and (2).

*Under the state of emergency: Please answer by taking the 18-month period from April 2020 to September 2021 as "under the state of emergency," although this period differs in part from the actual period of the declared state of emergency.

(1) Yes or no at present

(2) Under the state of emergency (April 2020–September 2021)

	(1)	(2)
1. A lot		
2. Somewhat		
3. Not much		
4. No		

Q19. Who do you talk to about your problems and stress? Please answer all that apply.

1. Family
2. Friends and acquaintances
3. Supervisors at work, teachers at school
4. Professional counselling services (doctors, government, NPOs, etc.)
5. Consultation corner of TV, radio, newspapers, etc.
6. Consult with a large number of people on social media
7. **Other**
8. I want to consult but am unable to do so.
9. I want to consult but do not know where to go for advice.
10. I don't consult with anyone because I don't need to.

Q20. Is there a department where you regularly go to have illness, injury, etc., treated since before the declaration of the state of emergency (before April 2020)? Please answer all that apply.

1. Pulmonology (treatment of diseases of the lungs, bronchial tubes, etc.)
2. Gastroenterology (treatment of diseases of stomach, intestines, liver, pancreas, etc.)
3. Cardiology (treatment of diseases of the heart, blood vessels, etc.)
4. Urology (treatment of diseases of kidney, ureter, etc.)
5. Internal medicine (treating infectious diseases such as colds and influenza, as well as general

internal diseases with medicine)

6. Surgery (surgical treatment of illness or injury)

7. Orthopedic surgery (treatment of diseases and injuries of bones, muscles, and other locomotory organs)

8. Dermatology

9. Psychosomatic medicine, psychiatry, neurology (treatment of mental illness)

10. Obstetrics and gynecology

11. Ophthalmology, otorhinolaryngology

12. Dentistry

13. Other (　　　　)

14. N/A

[For those with a department they regularly attend]

Q21. Did your illness, injury, or other condition change under the state of emergency (April 2020–September 2021)?

*Under the state of emergency: Please answer by taking the 18-month period from April 2020 to September 2021 as "under the state of emergency," although this period differs in part from the actual period of the declared state of emergency.

1. Improved under the state of emergency and still improved
2. Improved under the state of emergency, but now back to normal
3. Worsened under the state of emergency, but now back to normal
4. Deteriorated under the state of emergency and still in a deteriorated condition
5. No particular change

[For those with a department they regularly attend]

Q22. Did the frequency of your hospital visits change under the state of emergency (April 2020–September 2021)?

*Under the state of emergency: Please answer by taking the 18-month period from April 2020 to September 2021 as "under the state of emergency," although this period differs in part from the actual period of the declared state of emergency.

1. The frequency of hospital visits increased from before the state of emergency, and I am still visiting the hospital at that frequency.
2. The frequency of hospital visits increased from before the state of emergency, but has returned to the original frequency.
3. The frequency of hospital visits decreased from before the state of emergency, but has returned to the original frequency.
4. The frequency of hospital visits decreased from before the state of emergency, and I am still visiting the hospital at that frequency.
5. No particular change

[For those with children under the age of 18]

Q23. Did the frequency of your child's hospital visits change under the state of emergency (April 2020–September 2021)? If you have more than one child who visits the hospital regularly, please answer for the child who visits the hospital most frequently.

*Under the state of emergency: Please answer by taking the 18-month period from April 2020 to September 2021 as "under the state of emergency," although this period differs in part from the actual period of the declared state of emergency.

1. No child is a regular visitor to the hospital.
2. The frequency of hospital visits increased from before the state of emergency, and the child is still visiting the hospital at that frequency.
3. The frequency of hospital visits increased from before the state of emergency, but has returned to the original frequency.
4. The frequency of hospital visits decreased from before the state of emergency, but has returned to the original frequency.
5. The frequency of hospital visits decreased from before the state of emergency, and the child is still visiting the hospital at that frequency.
6. No particular change

Q24. Did you undergo any of the following health examinations under the state of emergency (April 2020–September 2021)? Please indicate for (1) and (2) which applies to you.

*Under the state of emergency: Please answer by taking the 18-month period from April 2020 to September 2021 as "under the state of emergency," although this period differs in part from the actual period of the declared state of emergency.

(1) Medical checkups and physical examinations

(2) Cancer screening

	(1)	(2)
1. Attended as usual		
2. Attended at a different time of the year from previous years		
3. Did not attend under the state of emergency (attended in previous years)		
4. Did not attend under the state of emergency (did not attend in previous years)		

Q25. Do you have a family doctor?

1. I have a family doctor
2. There is a doctor that comes to mind
3. No

4. Don’t know

Q26. Have you been vaccinated?

1. 2 inoculations
2. 1 inoculation
3. Not inoculated

[For those with a department they regularly attend]

Q27. Did you receive online medical care under the state of emergency (April 2020–September 2021)?

*Under the state of emergency: Please answer by taking the 18-month period from April 2020 to September 2021 as “under the state of emergency,” although this period differs in part from the actual period of the declared state of emergency.

1. Received online medical care under the state of emergency and continue to receive it
2. Received online medical care under the state of emergency, but not currently receiving it
3. Did not receive online medical care under the state of emergency, but now receiving it
4. Not receiving online medical care either under the state of emergency or at present

Q28. Would you like to receive online medical care in the future?

1. I would very much like to receive it.
2. I would like to receive it.
3. I don’t want to take on too much
4. I do not want to accept
5. Don’t know

Q29. (1) Please indicate all the things you currently do for your health.

(2). Please indicate all the areas for which you had difficulties under the state of emergency (April 2020–September 2021).

*Under the state of emergency: Please answer by taking the 18-month period from April 2020 to September 2021 as “under the state of emergency,” although this period differs in part from the actual period of the declared state of emergency.

	(1)	(2)
1. Balanced diet		
2. Moderate exercise (including sports) or physical activity		
3. I get sufficient sleep		
4. Not smoking cigarettes		
5. I try not to drink too much alcohol.		
6. I try not to be stressed		
7. Regularly undergo health checkups		

**8. Other**		
9. Nothing in particular		

Q30. Did your physical activity change under the state of emergency (April 2020–September 2021)?

*Under the state of emergency: Please answer by taking the 18-month period from April 2020 to September 2021 as "under the state of emergency," although this period differs in part from the actual period of the declared state of emergency.

1. My exercise increased under the state of emergency and has remained at that level
2. My exercise increased under the state of emergency, but has returned to normal
3. My exercise decreased under the state of emergency, but has returned to normal
4. My exercise decreased under the state of emergency and has remained at that level
5. No particular change

Q31. Did your alcohol consumption change under the state of emergency (April 2020–September 2021)?

*Under the state of emergency: Please answer by taking the 18-month period from April 2020 to September 2021 as "under the state of emergency," although this period differs in part from the actual period of the declared state of emergency.

1. My drinking increased under the state of emergency and has remained at that level
2. My drinking increased under the state of emergency, but has returned to normal
3. My drinking decreased under the state of emergency, but has returned to normal
4. My drinking decreased under the state of emergency and has remained at that level
5. No particular change
6. I do not drink alcohol

Q32. Did the number of cigarettes you smoke change under the state of emergency (April 2020–September 2021)?

*Under the state of emergency: Please answer by taking the 18-month period from April 2020 to September 2021 as "under the state of emergency," although this period differs in part from the actual period of the declared state of emergency.

1. The number of cigarettes I smoke increased under a state of emergency and has remained at that level
2. The number of cigarettes I smoke increased under the state of emergency, but has returned to normal
3. The number of cigarettes I smoke decreased under the state of emergency, but has returned to normal
4. The number of cigarettes I smoke decreased under the state of emergency and has remained at that level

5. No particular change
6. I do not smoke

Q33. Did your weight change under the state of emergency (April 2020–September 2021)?

*Under the state of emergency: Please answer by taking the 18-month period from April 2020 to September 2021 as "under the state of emergency," although this period differs in part from the actual period of the declared state of emergency.

1. I gained weight under the state of emergency, which I maintain to date
2. I gained weight under the state of emergency, but it has returned to normal
3. I lost weight under the state of emergency, but it has returned to normal
4. I lost weight under the state of emergency, which I maintain to date
5. No particular change

Q34. What are your daily measures against COVID-19? Please indicate this with (1) and (2) for each of the following.

(1) Actions currently being taken
(2) Actions to be continued even if the threat of infection is lowered

	(1)	(2)
1. Wearing a facemask		
2. Washing hands and gargling		
3. Disinfecting with alcohol		
4. Keeping your distance from others and avoiding crowded or enclosed areas		
5. Refraining from unnecessary and non-urgent outings		
6. Refraining from eating and drinking in large groups, for long periods of time, and at occasions that involve alcohol consumption		
7. Avoiding stores and places that do not have measures against COVID-19		
8. Regularly checking temperature		
**9. Other**		
10. Nothing in particular		

Q35. I would like to ask about policy for daily living with COVID.
What do you think of the idea of loosening restrictions on the activities of those at low risk of infection by having them presenting proof of vaccination or negative PCR test results, making it easier for them to use restaurants, travel, attend events, etc.?

1. I would like to see it applied in a wide range and variety of fields
2. I want it to be applied only to the minimum extent necessary

3. I don’t want it to happen
4. Don’t know

- About interaction and connections with friends

Q36. How often on average do you interact (meeting in person or keeping in touch) with your friends? Please indicate the frequency for (1) and (2).

*Under the state of emergency: Please answer by taking the 18-month period from April 2020 to September 2021 as “under the state of emergency,” although this period differs in part from the actual period of the declared state of emergency.

(1) Current frequency

(2) Average frequency under the state of emergency (April 2020–September 2021)

	(1)	(2)
1. Almost every day		
2. 3 or 4 times a week		
3. Once a week		
4. 2 or 3 times a month		
5. Once a month		
6. Less than once a month		
7. None of the above		

Q37. Do you have family or friends you can rely on when you are in trouble?

1. Family members and relatives living together
2. Family members and relatives other than those living together
3. Friends and acquaintances
4. Colleagues and supervisors at work
5. Boyfriend/girlfriend
6. School teacher (mentor)
7. None
8. **Other**

[For those who have someone to rely on other than a family member living with them]

Q38. Excluding family members and relatives living with you, how many people in total do you rely on when you are in trouble?

1. 1 person
2. 2 persons
3. 3 persons
4. 4 persons
5. 5 persons

6. 6 to 9 persons

7. 10 to 19 persons

8. 20 to 29 persons

9. More than 30 persons

Q39. How often do you currently participate in volunteer activities (including PTA activities), neighborhood associations, and other local community activities (including hobbies and sports)? Please indicate the frequency for (1) and (2).

*Under the state of emergency: Please answer by taking the 18-month period from April 2020 to September 2021 as "under the state of emergency," although this period differs in part from the actual period of the declared state of emergency.

(1) Current frequency of participation

(2) Average frequency of participation under the state of emergency (April 2020–September 2021)

	(1)	(2)
1. Almost every day		
2. 3 or 4 times a week		
3. Once a week		
4. 2 or 3 times a month		
5. Once a month		
6. Less than once a month		
7. Not participating		

Q40. How often do you currently use social media (Facebook, Twitter, LINE, Instagram, etc.)?

1. Almost every day
2. 3 or 4 times a week
3. Once a week
4. 2 or 3 times a month
5. Once a month
6. Less than once a month
7. I do not use social media

[For social media users]

Q41. How many hours a day do you currently use social media (Facebook, Twitter, LINE, Instagram, etc.)?

1. 30 minutes or less
2. About 1 hour
3. About 2 hours
4. 3–4 hours

5. About 5–6 hours
6. More than that

Q42. How many friends do you have with whom you interact on social media (Facebook, Twitter, LINE, Instagram, etc.)?
1. None
2. 1 to 9 persons
3. 10 to 19 persons
4. 20 to 29 persons
5. 30 to 39 persons
6. 40 to 49 persons
7. 50 to 99 persons
8. 100 to 300 persons
9. 301 persons or more

Q43. Do you feel that the development of the Internet, including social media, has strengthened your social connections, such as friendships and communities?
1. I feel my connection with society has been strengthened
2. Can't say either way
3. I do not think so

- About your personal life

Q44. Please indicate for (1) through (3) the activities you perform at home using computers, tablets, smartphones, and other digital communication devices. ~~**(Please select all that apply)**~~

*Under the state of emergency: Please answer by taking the 18-month period from April 2020 to September 2021 as "under the state of emergency," although this period differs in part from the actual period of the declared state of emergency.

(1) What you are currently engaged with
(2) What we did under the state of emergency (April 2020–September 2021)
(3) What would you like to enhance in the future?

	(1)	(2)	(3)
1. Work (main business)			
2. Work (side job)			
3. Academic work/studies (home study for school, learning for qualifications and culture, self-development, etc.)			
4. Health promotion through online courses and health management apps			
5. Watching videos, online streaming movies, etc. as a hobby			

6. Gaming			
7. Browsing the Internet, social media, etc.			
8. Information dissemination, posting and content creation on the Internet, social media, etc.			
9. Online drinking and social gatherings			
10. Activities such as meeting people, finding friends, etc.			
11. Online shopping			
12. Online meal ordering and delivery services (Uber Eats, etc.)			
13. Making listings on Internet shopping sites, auctions, etc.			
14. Online investment and asset management			
**15. Other**			
16. Nothing in particular			

Q45. How has the division of labor between husband and wife regarding household chores and childcare changed under the state of emergency (April 2020–September 2021)?

*Under the state of emergency: Please answer by taking the 18-month period from April 2020 to September 2021 as "under the state of emergency," although this period differs in part from the actual period of the declared state of emergency.

1. The husband started to do more
2. The husband started to do a little more
3. The wife started to do more
4. The wife started to a little more
5. Both the husband and wife started to do more
6. Both the husband and wife started to do less
7. No change in the division of labor between husband and wife

Q46. Please select the most important factor(s) in changes in the division of household chores and childcare between husband and wife.

1. The division of labor changed in the wake of the spread of COVID-19, but has largely returned to normal
2. The division of labor changed in the wake of the spread of COVID-19 and remains the same
3. There was no change in the division of labor with the spread of COVID-19
4. **Other**
5. Don't know

Q47. Approximately what is your household's total annual income (including taxes and social insurance premiums)?

1. Less than 1 million yen

2. 1 million yen or more but less than 3 million yen
3. 3 million yen or more but less than 5 million yen
4. 5 million yen or more but less than 7 million yen
5. 7 million yen or more but less than 10 million yen
6. 10 million yen or more but less than 20 million yen
7. 20 million yen or more but less than 30 million yen
8. 30 million yen or more but less than 50 million yen
9. 50 million yen or more but less than 1 billion yen
10. 1 billion yen or more

Q48. What is your annual income (including taxes and social insurance) from your own work?

1. Less than 1 million yen
2. 1 million yen or more but less than 3 million yen
3. 3 million yen or more but less than 5 million yen
4. 5 million yen or more but less than 7 million yen
5. 7 million yen or more but less than 10 million yen
6. 10 million yen or more but less than 20 million yen
7. 20 million yen or more but less than 30 million yen
8. 30 million yen or more but less than 50 million yen
9. 50 million yen or more but less than 1 billion yen
10. 1 billion yen or more
11. I don’t want to answer.

Q49. Approximately what are the financial assets (savings, securities, etc.) of your household as a whole (please provide the amount before subtracting debts, not net worth)?

1. Less than 1 million yen
2. 1 million yen or more but less than 3 million yen
3. 3 million yen or more but less than 5 million yen
4. 5 million yen or more but less than 7 million yen
5. 7 million yen or more but less than 10 million yen
6. 10 million yen or more but less than 20 million yen
7. 20 million yen or more but less than 50 million yen
8. 50 million yen or more but less than 1 billion yen
9. More than 1 billion yen but less than 3 billion yen
10. 3 billion yen or more

Q50(1) Please enter the name of the prefecture or municipality where you live.

Q50(2) Please enter your zip code.
